# Macrophages Orchestrate the Liver Tumor Microenvironment

**DOI:** 10.3390/cancers16091772

**Published:** 2024-05-04

**Authors:** Valeria Quaranta, Costanza Ballarò, Gianluigi Giannelli

**Affiliations:** National Institute of Gastroenterology, IRCCS “S. de Bellis” Research Hospital, Via Turi 27, Castellana Grotte, 70013 Bari, Italygianluigi.giannelli@irccsdebellis.it (G.G.)

**Keywords:** liver cancer, hepatocellular carcinoma, cholangiocarcinoma, tumor microenvironment, tumor-associated macrophages

## Abstract

**Simple Summary:**

Liver cancer is a deadly disease, in which hepatocellular carcinoma and cholangiocarcinoma are the most common types. Despite numerous advances, treatment options still remain poor for liver cancer patients. Tumor development and progression as well as response to treatment are highly modulated by the cellular and stromal components that together with tumor cells generate the tumor microenvironment (TME). Tumor-associated macrophages (TAMs) are one of the principal immune cell components of the liver TME, and they play a crucial role in sustaining tumorigenesis. This review summarizes recent findings on TAM biology, and it analyses the importance of the development of macrophage-targeting strategies for the treatment of liver cancer.

**Abstract:**

Liver cancer is one of the leading causes of cancer-related mortality. Hepatocellular carcinoma and cholangiocarcinoma are the most common types, and despite numerous advances, therapeutic options still remain poor for these cancer patients. Tumor development and progression strictly depend on a supportive tumor microenvironment (TME). Tumor-associated macrophages (TAMs) are the most abundant immune cells population within a tumorigenic liver; they sustain cancer cells’ growth and invasiveness, and their presence is correlated with a poor prognosis. Furthermore, TAM cross-talk with cells and components of the TME promotes immunosuppression, a desmoplastic response, and angiogenesis. In this review, we summarize the latest advances in understanding TAM heterogeneity and function, with a particular focus on TAM modulation of the TME. We also discuss the potential of targeting macrophage subpopulations and how this is now being exploited in current clinical trials for the treatment of liver cancer.

## 1. Introduction

Liver cancer is the fourth leading cause of cancer-related mortality [1]. Hepatocellular carcinoma (HCC) is the most common type and accounts for 80–90% of primary liver cancers. Cholangiocarcinoma (CCA) comprises a diverse group of tumors emerging in the biliary tree of the liver and includes intrahepatic cholangiocarcinoma (iCCA) and extrahepatic bile duct carcinoma (perihilar, pCCA, or distal cholangiocarcinoma, dCCA). CCA forms account for 6–15% of all primary liver cancers [2]. The incidence of other types of primary liver cancers, such as angiosarcoma or hepatoblastoma, is much lower [3]. 

The most important risk factor for developing liver cancer can be identified as chronic inflammatory processes. In particular, specific agents such as viral infections by hepatitis B virus (HBV) and C virus (HCV), metabolic alterations such as alcoholic steatohepatitis (ASH) and non-alcoholic steatohepatitis (NASH), and consequences of chronic toxin exposure such as aflatoxin or parasite infection (flukes, for example) have been considered risk factors associated with both HCC and iCCA [4,5]. Many risk factors for iCCA have been found to be similar to those known for HCC [6]. Some other risk factors are more specific to the cancer type or region [2]. For early-stage HCC, standard care treatments include resection, localized therapies such as ablation and radiation, and liver transplantation [7,8]. Unfortunately, the majority of patients present advanced or unresectable disease. Use of the multikinase inhibitors sorafenib and lenvatinib, which are the approved first-line systemic treatment options, only yield a modest prolongation of overall survival [9,10,11]. The prognosis for CCA patients is similarly poor. Indeed, CCA is often diagnosed when the disease is already in its advanced stages. In this case, cisplatin and gemcitabine chemotherapy are used as first-line treatments [12,13]. In liver cancer patients, immunotherapy is gaining a major role as a potential treatment strategy. However, although many efforts have been made to improve clinical benefit, patient response is critically limited by the presence of an immunosuppressive liver microenvironment, and the treatment is confined to patient subsets with high CD8^+^ T cell infiltration [14]. Numerous studies are now focusing upon evaluating the effect of synergistic approaches that target different mechanisms including vascular normalization to improve drug delivery and immune infiltration, the activation of anti-tumor immune responses, and/or suppression of immune cells with tumor-promoting functions [15]

The tumor microenvironment (TME) is the cellular environment in which the solid tumor exists, and it is composed of immune cells, cancer-associated fibroblasts (CAFs), tumor-related endothelial cells, extracellular matrix (ECM) components, and a milieu of metabolites, signaling molecules, and proteins in the intercellular space [3]. The TME is an active participant and a major regulator of key hallmarks of liver tumors, including altered energy metabolism, tumor-promoting inflammation, angiogenesis, invasion, and metastasis [16]. The component of the immune system in the TME is extremely heterogeneous, and it consists of infiltrating CD8^+^ and CD4^+^ T cells, T regulatory cells (T_regs_), natural killers (NKs), dendritic cells (DCs), macrophages, and neutrophils. The immune system has a dual and controversial relationship with cancer cells. Indeed, on one hand, the immune cells are responsible for the detection and elimination of nascent tumor cells; on the other hand, the immune system can become corrupted and promote cancer development and progression [17]. Macrophages are one of the immune cell populations that have attracted much attention in the recent years. They have an extremely heterogenous and plastic phenotype, and their diverse functions make them a key contributor to maintaining hepatic homeostasis and orchestrating tumor development and progression [18]. 

In this review, we highlight novel findings regarding the characterization of tumor-associated macrophages (TAMs)’ biology. In particular, we will focus on understanding how TAM cross-talk with the different liver TME components affects tumor development and progression and how the knowledge of TAMs’ heterogeneity and function can support the development of novel therapeutic strategies. 

## 2. Liver Macrophages

The liver is considered an immune organ, as in physiological conditions it has a unique microenvironment. Blood flood in the liver derives primarily from the gastrointestinal tract through the portal vein. Therefore, due to the constant liver targeting of gut-derived pathogens, immunosurveillance and an immunosuppressive microenvironment are crucial for maintaining self-tolerance and avoiding a severe autoimmune-response [19,20]. Macrophages are the most abundant immune cells type in the liver, and although different populations are present, they mainly consist of Kupffer cells (KCs) and monocyte-derived macrophages (MoMs) [21]. KCs are liver-residing macrophages that lie on liver sinusoids and play a key role in orchestrating the immune-tolerant orientation of the liver by expressing programmed cell death ligand 1 (PD-L1) and low levels of costimulatory molecules (CD80 and CD86) by priming T_reg_ and by secreting interleukin (IL)-10 and Transforming Growth Factor beta (TGFβ). Recently, it has been demonstrated that KCs originate from yolk sac-derived colony-stimulating factor receptor (CSF1R)^+^ erythromyeloid progenitors (EMPs) resident in the fetal liver during embryogenesis, which then differentiate into fetal monocytes and then KCs [22,23]. Additionally, yolk-sac EMPs may generate circulating precursors (pre-macrophages) that populate the embryo in a C-X3-C motif chemokine receptor (CX3CR)1-dependent manner and give rise to KCs upon colonization [24]. Single-cell RNA sequencing (scRNA-Seq) analysis performed by Sonya et al. on human livers revealed the presence of two distinct populations of CD68^+^ macrophages. One such population is that of inflammatory macrophages, which feature abundant markers such as LYZ, CSTA, CD74. The second population of CD68^+^ macrophages present differently expressed genes such as CD5L, MARCO, VSIG4, CD163, and LIPA, which suggests a macrophage tolerogenic function [25]. KCs self-renew throughout adult life with minimal replenishment from circulating cells [26,27,28]. However, it has been demonstrated that depletion of KCs in Clec4f-DTR transgenic mice leads to the recruitment of circulating monocytes into the liver followed by differentiation into KC-like cells (named monocyte-derived KC, moKC), which are transcriptionally similar to embryologically derived KCs (emKCs) [29,30]. Similarly, replenishment of emKCs by circulating monocytes could occur in a range of liver injuries such as viral hepatitis [31] or fatty acid liver disease, and their differentiation into moKCs is strictly dependent on signaling pathways such as Notch or TGF-β, activated by liver TME components [32,33,34,35]. KCs also act as first-line defense upon liver injury. They possess high phagocytic abilities and secrete different types of chemokines and cytokines in response to signals associated with the onset and progression of liver disease, including the release of oxygen species (ROS), damage-associated molecular patterns (DAMPs), pathogen-associated molecular patterns (PAMPs) [36]. KCs support expression of the hypoxia-inducible factor (HIF)-1α caused by a hypoxic liver microenvironment [37,38] and promote the spread of extracellular vesicles containing pro-inflammatory factors such as the microRNA-27 (miRNA-27) [39]. Importantly, KCs are a major source of C-C motif chemokine ligand (CCL)2, leading to the recruitment of CCR2^+^ monocytes into the diseased liver. In turn, the release of chemokines by the infiltrating monocytes attracts other immune cell components such as neutrophils, NKs, and T cells, which further contribute to exacerbate inflammation and fibrogenesis [40]. During tissue injury, the liver macrophage landscape drastically changes in a disease-associated manner, featuring a switch from a KC-dominant to a MoMs-dominant microenvironment. The liver of patients with alcoholic liver disease (ALD), NASH, primary biliary cholangitis, or primary sclerosing cholangitis is infiltrated by CD14^high^CD16^neg^ monocytes which secrete proinflammatory cytokines such as tumor necrosis factor (TNF)α, IL-1β, CCL1, and CCL2 [41]. Hepatic inflammation, fibrosis, and cirrhosis have been observed in 80% of HCC patients [5], whereas CCA often arises in the setting of prolonged biliary inflammation and/or cholestasis, which contribute to carcinogenesis [2]. In these settings, pro-inflammatory macrophages play a key role by providing a permissive microenvironment that favors the succession from chronic inflammation to tumor lesions initiation and progression [42].

## 3. TAMs in Liver Cancer

In the liver tumor, TAMs are believed to mainly derive from infiltrating monocytes that originate from the myelopoiesis of hematopoietic stem cell (HSC) precursors in the bone marrow. Mobilization of HSC-derived monocytes that reside in the spleen represents a secondary source of TAMs [43]. Furthermore, some evidence supports the view that tissue-residing macrophages also contribute in a small portion to the TAMs pool [44,45]. Monocytes are recruited in to the tumor site and differentiate into macrophages upon tissue infiltration, and this process is mediated by inflammatory signals such as CCL2, CCL5, and CSF-1 secreted by tumor cells and other cells within the malignant TME [36].

Macrophages are a highly plastic cell population whose gene expression profile changes depending on the surrounding TME. Inflammatory stimuli, including interferon- gamma (IFNγ) and microbial products such as lipopolysaccharide molecules (LPS) can induce a macrophage polarization toward an M1-like or ‘classical activated’ phenotype. M1-like macrophages are characterized by a high antigen-presenting capability and a high expression of pro-inflammatory cytokines such as IL-12 and tumor necrosis factor α (TNFα), which mediate the activation of a T helper 1 (Th1) immune response. Furthermore, M1-like macrophages trigger cytotoxic activity toward microorganisms and cancer cells by upregulating ROS and nitric oxide (NO). Conversely, the presence of T helper 2 (Th2)- related cytokines and growth factors such as IL-4, IL-13, IL-10 in the TME induces an alternative activation of macrophages, also known as M2-like macrophage phenotype. M2-like macrophages are characterized by poor antigen-presenting ability; expression of certain cytokines including IL-10, TGFβ, and CCL17; high expression of scavenger receptors such as CD163 and mannose receptor (MRC1/CD206); and high levels of PD-L1. As such, M2-like macrophages correlate with an anti-inflammatory phenotype and with ECM remodeling and immunosuppressive properties [46]. 

**Figure 1:**
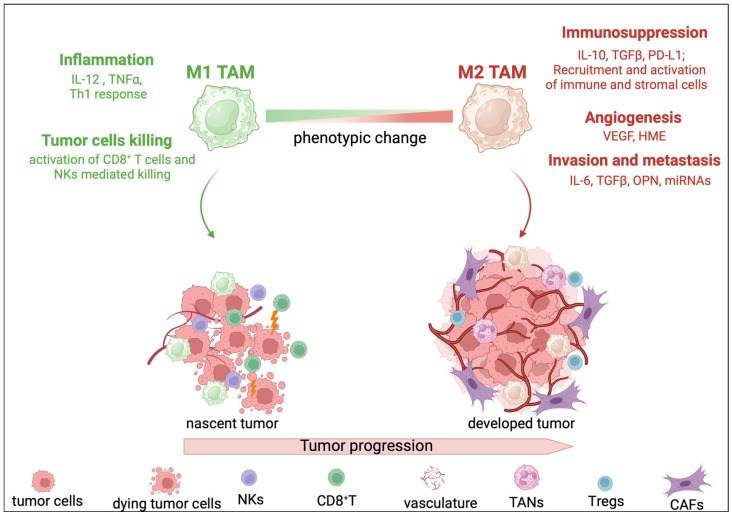
Phenotypic change in tumor-associated macrophages (TAMs) during tumor progression. Macrophages are a highly plastic immune cell population whose gene expression profile changes depending on the surrounding tumor microenvironment (TME). Generally, TAMs with a relatively M1-like skewed phenotype are activated during the early development of tumorigenesis and exert an anti-tumorigenic function. M1-like TAMs release pro-inflammatory cytokines, including interleukine (IL)-12 and tumor necrosis factor α (TNFα), thereby triggering a T helper 1 (Th1)-mediated immune response. This, in turn, leads to the activation and recruitment of cytotoxic CD8^+^ T cells that together with natural killer (NK) cells induce tumor cell death by the release of cytotoxic factors such as granzymes and perforin. However, tumors cells are able to corrupt surrounding cells in TME, including TAMs, and to induce them to acquire pro-tumorigenic features. Indeed, tumor progression correlates with high abundance of TAMs with an M2-like phenotype. M2-like TAMs play a key role in all the steps of tumor progression: they suppress the anti-tumorigenic function of cytotoxic immune cells through the secretion of immunosuppressive cytokines (IL-10 and tumor growth factor β (TGFβ), for example); the expression of immuno-checkpoint ligands, namely programmed cell death ligand 1 (PD-L1); and the recruitment of other cell types, such as T regulatory cells (T_regs_), tumor-associated neutrophils (TANs) and cancer-associated fibroblasts (CAFs). Furthermore, M2-like TAMs act as regulators of angiogenesis thought the secretion of growth factors such as vascular endothelial growth factor (VEGF) and human macrophage metalloelastase (HME). M2-like TAMs promote invasion and metastasis through the release of various cytokines including IL-6, TGFβ, osteopontin (OPN) and through the expression of non-coding RNA molecules such as microRNAs (miRNAs).

Epidemiological evidence indicates that TAMs are abundant in HCC tumors compared to the non-tumoral adjacent liver tissue [47], and their presence is correlated with an increased tumor recurrence rate and poor disease-free survival in CCA [18,48]. In the liver tumor, the signals that orchestrate macrophages’ phenotype and function can vary depending on the stage of tumor progression or even between the different parts of the same tumor, thereby prompting different TAM phenotypes. Generally, TAMs with a relatively M1-like skewed phenotype are involved in suppressing the early development of liver tumorigenesis by exerting a tumor-killing function and by taking part in the activation of the adaptative immune response orchestrated by CD8^+^ T lymphocytes [49]. To overcome the tumoricidal effects of the immune system, tumors cells are able to reshape the surrounding microenvironment and educate TAMs to acquire features more commonly associated with an M2-like phenotype. Indeed, M2-like TAMs are involved in the immunosuppression of cytotoxic CD8^+^ T cells and NKs through various mechanisms, including secretion of immunosuppressive cytokines, engagement of immuno-checkpoint molecules, and recruitment of other immune cells such as T regulatory cells (T_regs_), for example [50]. Furthermore, M2-like TAMs are active participants in all the tumor-progressing steps. They act as regulators of angiogenesis through the secretion of angiogenic growth factors such as vascular endothelial growth factor (VEGF) [51]. M2-like TAMs promote invasion and metastasis through the release of various cytokines including IL-6, TGFβ and osteopontin (OPN) [52,53], as well as through the expression of non-coding RNA molecules such as miRNAs [54] (Figure 1).

Nowadays, technologies such as single-cell transcriptomic, epigenomic, metabolic and spatial multi-omics have allowed us to investigate cellular heterogeneity in cancer and to identify significant diversity in TAMs. Indeed, it is acknowledged that the TAM phenotype cannot be simply explained by M1–M2 dichotomy, and multiple studies have observed the presence of mixed macrophage phenotypes in TAMs. For example, Zhang et al. identified six macrophage clusters, among which Mφ-c1-THBS1 and Mφ-c2-C1QA are enriched in HCC tumor tissue. Interestingly, the Mφ-c2-C1QA subpopulation simultaneously resembles TAM, M1, and M2 signatures, and it is characterized by the expression of a set of genes including APOE, C1QA, C1QB, and TREM2 [55]. Research conducted by Sharma et al. in HCC has identified different subpopulations of TAMs characterized by CD163^high^ and folate receptor beta (FOLR2) expression and other clusters of CD163^low^ TAMs characterized by the expression of Osteopontin (SPP1) or Metallothionein 1G (MT1G). Interestingly, the authors found that FOLR2^high^/CD163^high^ TAMs exhibit a strong similarity to fetal liver macrophages and are enriched in tumor tissue as compared to adjacent normal liver, indicating a fetal-like reprogramming of TAMs in HCC. In addition, FOLR2^+^ TAMs are associated with the expression of checkpoint receptors and ligands as well as the expression of chemokines like CXCL12 and CXCL16, thereby suggesting a role in facilitating an immunosuppressive microenvironment [45]. In another study, a TAM subset resembling scar-associated macrophages (SAMs) was identified in liver cancer [56]. SAMs have a gene signature expression of CD9, TREM2, CAPG, GPNMB and OLR1 that differentiates them from KCs [57]. ScRNASeq performed on iCCA patients reveals the presence of a macrophage subpopulation with immunosuppressive gene signatures including VEGFA, MMP19, S100A2, SIRPα, LAG3 in tumoral tissue as compared to para-tumoral tissue [58].

## 4. TAM Modulation of the Liver TME

**Figure 2:**
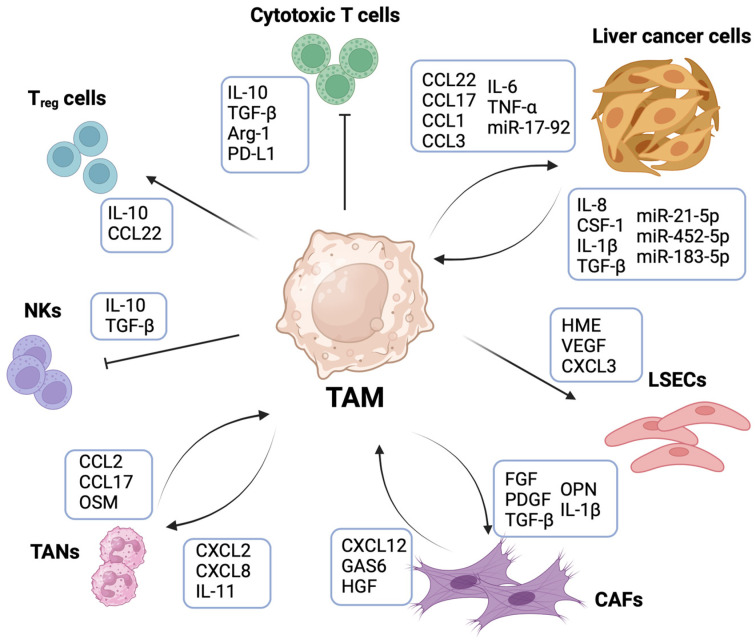
Tumor-associated macrophages (TAMs) orchestrate the liver tumor microenvironment (TME). TAM-mediated cross-talk with cells in the liver TME sustains tumor development and progression. TAMs support liver cancer cells growth and invasiveness through the release of several cytokines including C-C chemokine ligand (CCL)22, CCL17, CCL1, CCL3, interleukin (IL)-6, tumor necrosis factor (TNF)-α and macrophage-derived microRNA17-92 (miR-17-92). In turn, cancer cells promote TAM recruitment and a pro-tumorigenic and immunosuppressive TAM phenotype by the secretion of IL-8, IL-1β, tumor growth factor (TGF)-β, colony-stimulating factor (CSF)-1 and several tumor-derived microRNAs. TAMs promote liver sinusoid endothelial cells (LSECs)’ proliferation and neovascularization by expressing pro-angiogenic factors such as human macrophage metalloelastase (HME), vascular endothelial growth factor (VEGF) and C-X-C chemokine ligand (CXCL)3. Similarly, TAM cross-talk with cancer-associated fibroblasts (CAFs) is responsible for contributing to a desmoplastic reaction in tumorigenic liver. TAM-derived fibroblast growth factor (FGF), platelet-derived growth factor (PDGF), TGFβ, IL-1β and osteopontin (OPN) induce persistent activation of fibroblasts in liver cancer. CAF release of CXCL12, GAS6 and hepatocyte growth factor (HGF) promotes an M2-like TAM phenotype. TAMs further contribute to tumor progression by inducing the recruitment of other immune cells such as tumor-associated neutrophils (TANs) through the secretion of CXCL2, CXCL8 and IL-11. In turn, TANs contribute to macrophage recruitment by releasing CCL2, CCL17 and oncostatin M (OSM). TAMs induce the formation of an immunosuppressive microenvironment by inhibiting the cytotoxic activity of T cells and NKs. Indeed, TAMs are the cells with the highest expression of programmed cell death ligand 1 (PD-L1) within the TME, and they are the main source of the immunosuppressive molecules IL-10, TGFβ and Arginase-1 (Arg-1). TAM-derived IL-10 and CCL22 are also responsible for the recruitment of T regulatory cells (T_regs_), which further contribute to dampening immune cell-mediated anti-tumor response.

The heterogeneity and functional diversity of TAMs in liver cancer is influenced by the surrounding TME. The cross-talk of TAMs with cancer cells, liver sinusoid endothelial cells (LSECs), CAFs, and other immune cells types including neutrophils, T_regs_, NKs, and CD8^+^ T lymphocytes supports angiogenesis, fibrosis, immunosuppression, and ultimately, promotion of cancer growth and metastasis (Figure 2). 

### 4.1. TAMs and Tumor Cells’ Cross-Talk

Different signaling molecules orchestrate the cross-talk of TAMs with cancer cells, thereby affecting several processes involved in the tumor onset and progression. For example, HCC-secreted IL-8, IL-6, IL-1β, and CSF-1 [59,60,61,62] have been shown to promote TAM recruitment and a TAM immunosuppressive phenotype. Signaling pathways such as WNT and NOTCH are involved in TAM differentiation and therefore cancer progression [63]. Exosome-mediated cross-talk between HCC cells and TAMs has been revealed to regulate different phases of tumor initiation and progression through the release of miRNAs and long non-coding RNAs (lncRNAs). Tumor cell-derived miR-21-5p and miR452-5p are involved in TAM differentiation [64,65], whereas exosomal miR-17-92 derived from TAMs increases HCC cancer’s stemness properties and invasiveness [66]. In addition, it has been shown that tumor-derived exosomal miR-183-5p upregulates PD-L1-expressing macrophages to foster immune suppression in iCCA [67]. lcnRNAs instead are a class of RNA molecules which do not codify for proteins, but they function as biological signals, guides, and scaffolds [68]. TAM-expressed lncRNA H19, for example, has been found to promote HCC progression by regulating the miR-193b/MAPK1 axis [69]. 

It has also been shown that M2 macrophages promote HCC invasiveness through CCL22 secretion [70]. Activation of the WNT/β catenin pathway is involved in epithelial to mesenchymal transition (EMT) through the release of TNFα and CCL17 [71,72]. Consistently, macrophages are responsible for activation of the WNT pathway in CCA [73]. TAMs in iCCA promote EMT through the secretion of various cytokines and chemokines including IL-6, CCL1, CCL3 and the activation of AKT3/PRAS40 signaling [74]. Additionally, it has been shown that cholangiocarcinoma cells regulate TAM polarization and TGF-β1 secretion via the paracrine SHH signaling pathway. In turn, TAMs induce growth, EMT, and ER homeostasis in cholangiocarcinoma cells via TGF-β1 [75]. Evidence in a mouse model of CCA has demonstrated that M2-polarized macrophages promote tumor growth and invasiveness through IL-10/STAT3 signaling [76].

### 4.2. TAMs and Immune Cells’ Cross-Talk

TAMs exhibit close interactions with other immune cells within the TME. Notably, TAMs play a key role in immunosuppression and immune escape by regulating the activity of T cells [77]. TAMs release several immunosuppressive cytokines including IL-10, TGFβ, arginase-1 [3], and the expression of other molecules such as Singlec-10 and MARCKS has also been associated with poor prognosis in HCC [78,79]. Additionally, TAMs affect T cell cytotoxicity by interacting with co-stimulatory T cell molecules such as Ig-like transcript 2 (ILT2) and CD94 [80] as well as by expressing receptors of inhibitory molecules including PD-1, CTLA-4 and Tim3 [81]. TAMs are the major non-parenchymal cells that express PD-L1 [82], and PD-1^Hi^ CD8^+^ T cells are significantly enriched in HCC tumoral tissues compared to adjacent non-tumoral liver tissues [83]. In particular, it has been shown in HCC that IL-6 upregulates miR-25-3p through STAT3/c-MYC signaling, thereby promoting PD-L1 expression in macrophages [84]. Similarly, tumor-cell-derived exosomal miR-23a-3p has been reported to induce PD-L1 expression in TAMs and the consequent inhibition of T cell function [85]. TAMs are also a primary source of PD-L1 in human and murine CCA [86]. However, the presence of a mechanism compensating TAM blockage has been reported, and the failure to hinder tumor progression has been shown to be mediated by granulocytic myeloid-derived suppressor cells (GM-MDSCs), which are responsible for dampening the T cell response [87]. 

Macrophage secretion of cytokines, including CCL22, attracts T_reg_ to the tumor microenvironment, thereby hampering cytotoxic T cell activation and promoting tumor development [88]. It has been demonstrated that TREM-1^+^ TAMs respond to hypoxia and tumor metabolites via the ERK/NF-kb pathway, leading to the accumulation of CCR6^+^ Foxp3^+^ T_reg_ and promoting HCC resistance to PD-L1 treatment [89]. Moreover, Zhou et al. demonstrated an association of a high intra-tumoral density of FoxP3^+^ T_reg_, with a high density of TAMs dependent on IL-10 secretion [90]. 

In addition to dampening cytotoxic T cell activity, it has been shown that secretion of IL-10 and TGFβ by TAMs affects the cytotoxic activity of NKs [91] and that CD48 expression by macrophages induces NK cells’ dysfunction [92].

Mucosal-associated invariant T (MAIT) cells are MR1-restricted innate-like T cells that recognize non-peptide antigens including derivatives of microbiota-derived vitamin B2 (riboflavin) precursors. MAITs are an overabundant T cell subtype in the healthy human liver. The role of MAIT cells in cancer is not well defined, as increased MAIT cell numbers have been correlated with a poor prognosis in HCC [93]. In contrast, higher MAIT cell infiltration was correlated with a favorable prognosis within a cohort of CCA patients [94]. Another study identified PD-L1^+^ TAMs–MAIT cell interaction and demonstrated that αPD-1/αPD-L1 immuno-checkpoint blockade (ICB) reverses MAIT cells’ dysfunctionality in co-culture studies and murine models of HCC [95]. 

TAM interactions with other immune cells such as tumor-associated neutrophils (TANs) could also favor tumor progression. One such example is in the case of iCCA, in which TAN–TAM cross-talk mediated by oncostatin M and IL-11 has been shown to enhance the proliferation and aggression of the tumor though STAT3 signaling [96]. Similarly, monocyte-derived CXCL2 and CXCL8 are involved in neutrophils’ recruitment in HCC [97], whereas CCL2 and CCL17 secreted by TANs and peripheral blood neutrophils induce macrophage recruitment in the TME of HCC [98].

### 4.3. TAMs and LSECs’ Cross-Talk

LSECs play an active role in contributing to the TME and development of primary liver cancer. LSECs reside within sinusoids, and due to the expression of co-inhibitory molecules, they are responsible for inducing cytotoxic T cells’ tolerance [99]. Moreover, the secretion of tumor chemokines such as CXCL10, CCL2, CCL3, and adhesion molecules such as ICAM-1 and VAP-1 [100] by LSECs in HCC promotes leukocytes’ recruitment and the accumulation of other immunosuppressive cells like TAMs and T_regs_ [101]. It has been demonstrated that LSEC-derived plasmalemma vesicle-associated protein (PLVAP) regulates the egress of fetal liver monocyte-derived macrophages and their seeding in the tissue [102]. In support of this, research by Sharma et al. showed that HCC tumor tissue is particularly enriched by fetal-liver-associated PLVAP+ endothelial cells, and this is correlated with the presence of the fetal-liver-associated FOLR2^high^ TAMs subpopulation, supporting the hypothesis that tumor cells recapitulate the signature of early fetal development [45].

In liver cancer, the overexpression of pro-angiogenic factors regulates and promotes endothelial cell proliferation and neovascularization, thereby providing oxygen and nutrient components that support tumor growth. The Tie-2 macrophage/monocyte subpopulation mainly aggregates in the perivascular area of tumor tissue and participates in HCC angiogenesis. In particular, human macrophage metalloestase (HME) and VEGF have been implicated in angiogenesis [51,103]. In CCA patients, circulating CD14^+^CD16^+^ monocytes express Tie2 and high levels of growth and angiogenic factor-related genes such as VEGF-A, epiregulin, and CXCL3 [104]. CCR2^+^ TAMs have been found to be abundant at the edge of highly vascularized HCC [105], while CD14^+^ inflammatory macrophages have been found to secrete high levels of IL-23 upon stimulation by hepatitis virus-infected hepatocytes, and this is correlated with macrophage-induced angiogenesis [106].

### 4.4. TAMs and CAFs’ Cross-Talk

CAFs are a heterogenous population of cells that contribute to tumor progression in many cancers [107]. Consistently, CAFs are associated with aggressive tumor behavior and poor prognosis in patients with HCC and CCA [108,109]. CAFs originate from different cell types including mesenchymal progenitors such as hepatic stellate cells (HSCs), portal fibroblasts, vascular fibroblasts, and bone-marrow derived mesenchymal stem cells. CAFs in the liver are predominantly constituted by HSCs, while MYH11, ACTA2, THY1 and TAGLN have been described as CAF markers [110]. Numerous studies have shown that CAFs are involved in promoting liver cancer cells’ survival, proliferation, invasion, angiogenesis, immune escape and drug resistance [45]. Persistent activation of fibroblasts in cancer is mainly induced by TGFβ, fibroblast growth factor (FGF) and platelet-derived growth factor (PDGF) released by TAMs and tumor cells [111]. In CCA, fibroblast activation protein (FAP) ^+^ CAFs promote myeloid-derived suppressor cell infiltration [112], whereas the stromal-derived factor (SDF)1a-CXCR4 pathway is involved in CAF-mediated monocytes’ infiltration and differentiation within HCC tumors [113]. CAFs also contribute to M2 macrophage polarization by interacting with the CD68 receptor [114], and the CAF–TAM interactions result in an increased EMT in HCC. Indeed, CAFs promote M2 polarization of macrophages and induce plasminogen activator inhibitor-1 (PAI-1) secretion via CXCL12. PAI-1 produced by TAMs enhances tumor progression and ultimately metastasis [115]. Cross-talk between the liver macrophage subpopulation of SAMs and CAFs through WNT5 and hepatocyte growth factor (HGF) has been found to promote cancer and metastasis [57]. Liu et al. combined spatial transcriptomic analysis with scRNA-Seq analysis to reveal the presence of a specific structure, named the tumor immune barrier (TIB), in the TME of HCC patients who did not respond to immunotherapy. The research demonstrated that TIB is formed by the interaction of SPP1^+^ macrophages and CAFs. Interestingly, SPP1^+^ macrophages showed high TGFB1, SPP1, and IL1B ligand activity. The authors found that TGFB1 binds to TGFBR1, TGFBR2, and TGFBR3 expressed on CAFs, while SPP1 interacts with ITGA4, ITGA9, ITGAV, ITGB1, and ITGB5 on CAFs, and the ligand of IL1B on CAFs is IL1R1. As a result, collagen (COL1A1, COL1A2, COL3A1, COL4A1, and COL5A1), matrix metalloproteinase (TIMP1 and MMP), and chemokine (CCL3, 4, 5, and CXCR4) target genes were expressed in CAFs, leading to increased desmoplastic reactions in HCC. Moreover, the research showed that preclinical blockade of Spp1 destroys TIB and sensitizes HCC to immunotherapy [116]. These results are key steps that emphasize the importance of CAF–TAM interactions within the TME, thereby underlining the impact of targeting the TAM component in combination with ICBs.

## 5. Metabolic Regulation of TAMs

There is increasing evidence that reprogrammed energy metabolism of TAMs shapes their phenotype, thus playing a key role in the regulation of TME milieu and in contributions to cancer progression [117]. 

Tumor cells preferentially activate a metabolic pathway of aerobic glycolysis under normoxic conditions in order to rapidly increase adenosine triphosphate (ATP) energy levels, to increase macromolecules’ synthesis, and to prevent oxidative stress [118]. This process, also known as the Warburg effect, produces lactate and is recognized as an hallmark of cancer [119]. The Warburg effect has also been discovered to induce a TAM polarization switch toward a pro-tumorigenic and immunosuppressive phenotype. Indeed, lactate induces the release of VEGFA as well as arginase 1 (ARG1) and arginase 2 (ARG2), which dampen the activity of other immune cells and ultimately accelerate tumor progression [120]. It has been also shown that inhibition of aerobic glycolysis leads to reduced expression of M2 markers such as CD206, CD301, and CD163 [121]. In HCC, TAM expression of a high level of carbonic anhydrase XII (CA12) is associated with the presence of an M2-like TAM phenotype induced by hypoxia-inducible factor α (HIF-α), thus favoring tumor progression [122]. Other metabolic pathways are involved in the phenotypic switch of TAMs to an immunosuppressive phenotype. Lipid metabolism, in particular fatty acid oxidation (FAO), is crucial for the cellular metabolism. In fact, fatty acids are a fundamental cellular energy source, and they serve as constituents of the membrane structure [123]. Upregulation of genes linked to lipid metabolism has been found in TREM2^+^ and SPP1^+^ macrophages [124,125]. It has been also demonstrated that Sirtuin 4 (SIRT4) plays a role in lipid metabolism as its downregulation in TAMs modulates the alternative activation of macrophages and promotes HCC development via the FAO-PPAR δ-STAT3 axis [126].

Amino acid metabolism plays a key role in determining the macrophages’ phenotype and function. Glutamine, for example, is crucial for the synthesis of non-essential amino acids and nucleotides. An M2-like macrophage phenotype has been associated with elevated levels of glutamine metabolism, glutamine transporter proteins, and metabolic enzyme expression [127]. Arginine catabolism generates ornithine, which serves as a precursor for several biological molecules. Interestingly, both M1-like and M2-like macrophages utilize arginine through different mechanisms. iNOS is employed by M1-like macrophages to convert arginine into NO, which has an anti-tumor effect. Conversely, ARG1 and ARG2 are the enzymes used by M2-like macrophages to metabolize arginine, therefore leading to a decrease in NO production and an increase in the pro-tumorigenic effect [117]. Tryptophan metabolism also plays a role in the immunosuppressive function of TAMs as indoleamine 2,3-dioxygenase (IDO) can oxidize tryptophan in TAMs, leading to the generation of metabolites that suppress T cell functions [128]. Similarly, expression of the immunosuppressive macrophage molecule TREM2 correlates positively with purine metabolism and induces reduced levels of antigen-presentation-related proteins such as the major histocompatibility complexes (MHCs) I and II [129]. 

A correlation between the phenotype of TAMs and the pH level of the surrounding microenvironment has been found. Indeed, the presence of lactic acid contributes to the establishment of an acid milieu that fosters tumor progression. In the case of HCC, TAMs have the ability to adapt and survive the acidic environment by producing the vacuolar-type ATPase (V-ATPase); indeed, its inhibition induces a shift in TAMs toward an anti-tumorigenic type, accompanied by the upregulation of inflammatory cytokines [130].

Taken together, this evidence fosters the idea that modulation of metabolic pathways and related enzymes represents another approach to regulating the macrophage phenotype, thereby facilitating an anti-tumor response. 

## 6. Microbiome Regulation of TAMs

It is acknowledged that gut-microbiome-derived metabolites not only regulate local immunity in the intestine but also profoundly influence the immune system via host receptors and other target molecules [131]. Generally, gut microbial metabolites support immunity and tolerance, both of which are essential to maintain homeostasis and prevent chronic infection and inflammatory diseases. Pathological conditions can alter the production of microbial metabolites, leading to the dysregulation of the immune system and its metabolism [132]. Indeed, increased translocation of intestinal bacteria is a hallmark of chronic liver disease, and it contributes to hepatic inflammation and fibrosis [133]. Gut-derived endotoxins, such as PAMPs and LPS, trigger inflammatory responses through Toll-like receptors (TLRs) expressed on liver macrophages [134]. Other bacterial metabolites act as regulators of the function of hepatic macrophages. For example, microbiota-derived tryptophan metabolites reduce the pro-inflammatory abilities of macrophages [135], and a similar effect has been associated with the production of indole, another bacterial metabolite [136]. Certain microbial metabolites, such as short-chain fatty acids, are involved in regulating the activation of macrophages by G-protein-coupled receptor (GPCR) binding [137]. Butyrate (C4) derived from the Firmicutes phylum suppresses production of inflammatory cytokines, such as TNF-α, MCP-1, and IL-6 in macrophages [138]. 

Together these data suggest that advances in knowledge of the effect of gut microbial metabolites on TAMs, and more generally on the immune system, could promote the development of novel diagnostic, prognostic, and therapeutic modalities for liver diseases.

## 7. The Epigenetic Regulation of TAMs

Epigenetics refers to the alteration of gene expression without inducing changes in the DNA sequence. 

The mechanisms that regulate epigenetics involve alteration of gene accessibility for the transcription machinery, modification of the chromatin structure, and post-transcriptional alteration of the gene expression mediated by non-coding RNA molecules [139]. These modifications are mediated by numerous factors that include DNA methylation, histone modifications, chromatin remodeling, and interference in non-coding RNA-mediated gene expression. Studies carried out to understand the role of epigenetic modifications have mainly focused on cancer cells; however, more recent insights have shed light on the role of epigenetic modification in the development of a tumor-favorable TME. In particular, it has been shown that epigenetic alterations play a key role in the differentiation and functional reprogramming of TAMs in the context of liver cancer [140,141]. One study demonstrated that expression of CSF1R, which is essential for the survival of TAMs, is regulated by DNA methylation in HCC [142]. SIRT1, nicotinamide adenine dinucleotide (NAD)-dependent histone deacetylase has been shown to induce an M1-like polarization via the NF-kB pathway, thereby suppressing HCC metastasis [143]. A study performed by Zhang and colleagues has shown that lactate-derived lactylation of histone lysine residues serves as an epigenetic modification that directly stimulates gene transcription from chromatin. The authors demonstrated that polarization of bone marrow-derived macrophages (BMDMs) toward an M1-like phenotype stimulates histone lactylation and induces the expression of genes involved in wound healing, including Arg1, thus suggesting a progressive phenotypic switch of macrophages to an M2-like phenotype [144]. Similarly, histone lactylation is promoted by the acetyltransferase enzyme p300, thus favoring the activation of pro-wounding genes and a pro-tumorigenic macrophage phenotype [145].

Epigenetic modifications induced by miRNAs and lncRNAs also regulate macrophages’ phenotype and function [140]. Lnc cox-2, for example, promotes HCC metastasis by favoring the immune evasion of cancer cells and an M2-like macrophage polarization [146]. Different types of miRNAs promote epigenetic alterations. miR-98 influences the phenotypic switch from M2 to M1 by targeting IL-10 and suppressing TAM-mediated EMT in HCC [147]. Targeting of IL-34 by miR-28-5p suppress HCC progression and metastasis due to a reduced TAM infiltration [148]. Additionally, it has been shown that epigenetic silencing of the miR-144/451a cluster contributes to HCC progression via paracrine hepatocyte growth factor (HGF)/macrophage migration inhibitory factor (MIF)-mediated TAM remodeling [149].

## 8. Targeting TAMs

TAMs orchestrate different processes and interact with multiple cell types in the TME, and so targeting TAMs is considered one of the most promising treatment strategies for liver cancer. TAM-targeting strategies involve inducing the inhibition of their recruitment, depletion, and reprogramming (Table 1). In addition, considering the stringent interactions between TAM and other TME components including T cells, TAM-targeting strategies are often considered in combination with immunotherapy. The CSF-1/CSF1-R signaling axis is responsible for regulating macrophage survival and differentiation. Different types of drugs targeting the CSF-1/CSF1R axis have been tested in clinical trials for several cancer types, including the use of monoclonal antibodies and small-molecule inhibitors [150]. A phase 2 clinical trial (NCT04050462) with the anti-CSF1R antibody Cabiralizumab or BMS-986253, an IL-8 inhibitor, in combination with the PD-1 blocker Nivolumab is currently ongoing for advanced HCC patients. The use of Regorafenib, a blocker of multiple protein kinases, including CSF1R, in combination with Nivolumab is currently involved in a trial for improving outcomes for HCC patients based on the synergistic effects of the drugs (NCT04170556). Axatilimab (SNDX-6352), an inhibitor of CSF1R, in combination with Durvalumab following chemo–radio-embolization is undergoing phase II trials for unresectable iCCA (NCT04301778). CXCR4 is expressed on the macrophage surface, and it is involved in their tumor recruitment as well as in CAF interactions, desmoplasia, and T cell infiltration through the binding of its ligand, CXCL12. Therefore, inhibition of CXCL12-CXCR4 using AMD3100 in combination with Sorafenib and PD-1 blockade treatment showed an anti-tumor effect in a mouse model of HCC [151,152]. Similarly, combination of the CXCR4 antagonist, BPRCX807, with Sorafenib or anti-PD-1 prolonged overall survival in a mouse model of HCC [152,153]. Inhibition of the CCL5/CCR5 or CCL2/CCR2 axis is considered a therapeutic target for inhibiting macrophage recruitment. A study of the effect of administering Nivolumab with a CCR2/5-inhibitor or anti-IL8 (BMS-986253) before or after surgery is undergoing evaluation as a means of improving survival in HCC patients (NCT04123379).

Reprogramming the macrophages’ phenotype is a potent approach to reshaping their immunosuppressive abilities. CD47 is a ligand expressed in many tumor cell types, and by binding with the macrophage receptor CD47-signal regulatory protein alpha (SIRPα), it can regulate the phagocytic activity of macrophages. CD47 expression in HCC is correlated with poor overall survival of patients with HCC [154]. In addition, anti-human SIRPα antibodies have been developed for the treatment of HCC (NCT02868255). M2-to-M1 macrophage reprogramming by targeting STAT6 signaling using CDK-004, an anti-sense oligonucleotide (ASO), is currently under evaluation in a phase 1 clinical trial for the treatment of advanced HCC (NCT05375604). Similarly, PI3Kγ is a molecular switch involved in promoting immunosuppressive programs in TAMs [155]. Its inhibition by SF1126 inhibitor is under study in a clinical trial for liver and intrahepatic ductal carcinoma (NCT03059147). TLRs are also attracting attention as therapeutic approaches, because they play a crucial role in inducing an inflammatory response upon macrophage recognition of foreign antigens. Thus, the agonist approach for targeting TLR7/8 (RO7119929) is under consideration for HCC and biliary tract cancer (NCT04338685). The transcription factor CCAAT/enhancer-binding protein alpha (C/EBPα) is involved in the differentiation of myeloid cells as well as in their proliferation, metabolism, and immunity. In advanced HCC patients, treatment with the RNA oligonucleotide C/EBPα (saRNA; MTL-CEBPA), in combination with Sorafenib (NCT02716012), causes a reduction in tumor progression correlated with a marked decrease in peripheral blood monocytic myeloid-derived suppressor cell numbers and an overall reduction in numbers of pro-tumoral M2 TAMs [156]. Clinical trials using MTL-CEBPA in combination with chemotherapy and VEGF-A inhibitor (NCT05097911) or with anti-PD1 for solid tumor (NCT04105335) are currently ongoing.

CAR-T therapy is considered a revolutionary technique for cancer immunotherapy, but low infiltration of T cells is a major limitation. Since macrophages are one of the most abundant infiltrating cell types within the tumor, CAR macrophages (CAR-M) have emerged as a therapeutic strategy. CAR-Ms demonstrated antigen-specific phagocytosis and expression of a proinflammatory signature, leading to tumor cells’ clearance in solid tumor xenograft mouse models [157]. A first-in-human CAR m phase 1 clinical trial is ongoing for HER2-overexpressing solid tumors, including HCC and biliary tract neoplasms (NCT04660929).

## 9. Conclusions

In the liver, the role of TAMs not only depends on close interactions with tumor cells; it is also sustained and promoted by surrounding stroma cells, which influence their recruitment and polarization toward a pro-tumorigenic phenotype. In turn, TAM cross-talk with immune cells, endothelial cells, and fibroblasts contributes to the generation of an immunosuppressive and desmoplastic TME, which further promotes tumor development and progression. The plethora of functions and interconnections in which TAMs are involved makes them one of the main promising targets for the development of anti-tumor therapies. However, hepatic macrophages’ heterogeneity, their plastic phenotype, and their key role in maintaining liver homeostasis render them a challenging cell population for pharmacological targeting. In recent years, the use of technologies such as scRNASeq, spatial transcriptomics, and advanced imaging techniques have allowed for more in-depth characterization of macrophage populations based on tumor type, origin, and localization. This is leading to the development of more refined therapeutic strategies aimed at targeting tumor-specific TAM-expressing molecules, often conceived as a synergistic approach with first-line chemotherapy or ICB options. Despite numerous advances, we are still in the process of understanding liver TME complexity, cellular interactions, and response to therapy. In the future, improved pre-clinical models and further technological developments are expected. For example, more in-depth multi-omics analysis with broader applications to clinical samples will allow us to shed further light on liver macrophage biology and to develop personalized and targeted interventions.

## Figures and Tables

**Table 1:** Clinical trials of TAM-targeting agents.

TAM Target	TAM Targeting Agent	Combination Therapy	Liver Cancer Type	ClinicalTrial.Gov Reference
TAM depletion				
CSF-1/CSF-1R	anti-CSF-1R mAb (Cabiralizumab)	anti-PD-1 mAb (Nivolumab)	HCC	NCT04050462
	multi-protein kinase inhibitor (Regorafenib)	anti-PD-1 mAb (Nivolumab)	HCC	NCT04170556
	anti-CSF-1R mAb (SNDX-6352)	anti-PD-L1 mAb (Durvalumab)	iCCA	NCT04301778
Inhibition of TAM recruitment				
CCR2/CCR5	CCR2/CCR5 inhibitor (BMS-813160)	anti-PD-1 mAb (Nivolumab)	HCC	NCT04123379
TAM reprogramming				
CD47/SIRPα	anti-hu SIRPα Ab	N/A	HCC	NCT02868255
STAT6	exoASO-STAT6 (CDK-004)	N/A	HCC	NCT05375604
PI3Kγ	PI3Kγ inhibitor (SF1126)	anti-PD-1 mAb (Nivolumab)	HCC	NCT03059147
TLR7/TLR8	TLR7 agonist (RO7119929)	N/A	HCC and biliary tract cancer	NCT04338685
C/EBPα	Small activating RNA (MTL-CEBA)	Kinase inhibitor (Sorafenib)	HCC	NCT02716012
		Chemotherapy and VEGF-A inhibitor	HCC	NCT05097911
		anti-PD-1 mAb (Pembrolizumab)	HCC and biliary tract cancer	NCT04105335
HER2	CAR-M	N/A	HCC and biliary tract cancer	NCT04660929

Clinical trials of tumor-associated macrophage (TAM)-targeting agents. Data were obtained from https://classic.clinicaltrials.gov (accessed on 20 March 2024). CSF-1, colony-stimulating factor; PD-1, programmed cell death protein-1; CCR, C-C chemokine receptor; exoASO, Anti-sense oligonucleotide; SIRPα, CD47 signal-regulatory protein alpha; TLRs, Toll-like receptors; C/EBPα, CCAAT/enhancer-binding protein alpha; CAR-M, chimeric antigen receptor macrophages; N/A, not applicable.
